# An efficient system for intestinal on-site butyrate production using novel microbiome-derived esterases

**DOI:** 10.1186/s13036-021-00259-4

**Published:** 2021-03-06

**Authors:** Dah Hyun Jung, Ji Hyun Yong, Wontae Hwang, Mi Young Yoon, Sang Sun Yoon

**Affiliations:** 1 Department of Microbiology and Immunology, Yonsei University College of Medicine, 50-1 Yonsei-ro, Seodaemun-gu, Seoul, 03722 South Korea; 2 Brain Korea 21 PLUS Project for Medical Sciences, Yonsei University College of Medicine, Seoul, Republic of Korea; 3 Severance Biomedical Science Institute, Yonsei University College of Medicine, Seoul, Republic of Korea; 4 Institute for Immunology and Immunological Diseases, Yonsei University College of Medicine, Seoul, Republic of Korea

**Keywords:** Tributyrin, Butyrate, Esterase, Prodrug, Microbiome, Metagenomic library, Colitis, Inflammatory bowel disease (IBD), Bacteroidales, Clostridiales

## Abstract

Short-chain fatty acids, especially butyrate, play beneficial roles in sustaining gastrointestinal health. However, due to limitations associated with direct consumption of butyrate, there has been interest in using prodrugs of butyrate. Tributyrin (TB), a triglyceride composed of three butyrate molecules and a glycerol, is a well-studied precursor of butyrate. We screened a metagenome library consisting of 5760 bacterial artificial chromosome clones, with DNA inserts originating from mouse microbiomes, and identified two clones that efficiently hydrolyse TB into butyrate. Nucleotide sequence analysis indicated that inserts in these two clones are derived from unknown microbes. BLASTp analysis, however, revealed that each insert contains a gene homologous to acetylesterase or esterase genes, from *Clostridium* spp. and *Bacteroides* spp., respectively. Predicted structures of these two proteins both contain serine-histidine-aspartate catalytic triad, highly conserved in the family of esterases. *Escherichia coli* host expressing each of the two candidate genes invariably produced greater amounts of butyrate in the presence of TB. Importantly, administration of TB together with cloned *E. coli* cells alleviated inflammatory symptoms in a mouse model of acute colitis. Based on these results, we established an efficient on-site and real-time butyrate production system that releases butyrate in a controlled manner inside the intestine.

**Supplementary Information:**

The online version contains supplementary material available at 10.1186/s13036-021-00259-4.

## Introduction

Our immanent symbiont, the intestinal microbiome, has been intensely studied due to its realised impact on human physiology. Dysbiosis of commensal bacteria and alteration of commensal-derived metabolites in the gut trigger greater vulnerability to pathogenic infections, inappropriate immune response or even systematic disorders [1]. Among various bacterial metabolites produced in the gut, short chain fatty acids (SCFAs) are mainly produced by anaerobic fermentation of indigestible polysaccharides by commensal anaerobes, and SCFAs have been highlighted as a key factor for sustaining gut homeostasis [2]. In particular, butyrate, one of the major SCFAs in the gastrointestinal tract, serves as a major energy source for colonocytes and also a signalling molecule as histone deacetylase inhibitor and a ligand of GPR41 or GPR43 receptor [3, 4]. Ultimately, butyrate can enhance the gut barrier function of intestinal epithelial cells, exert anti-inflammatory effects, and suppress the occurrence of colorectal cancer [5–7].

Inflammatory bowel disease (IBD), of which major subtypes are ulcerative colitis (UC) and Crohn’s disease (CD), is a chronic and relapsing inflammatory condition in the gastrointestinal tract accompanied by persistent diarrhoea, abdominal pain, rectal bleeding, and weight loss [8]. Though the exact trigger of IBD remains unclear, many research indicate that IBD is a result of inappropriate immune response in genetically susceptible individuals [9].

Emerging evidence strongly suggests that dysbiosis of the gut microbiome and IBD are highly correlated, demonstrated by the compositional differences between the stool samples of IBD patients and healthy controls [10]. A significant change of microbial diversity in the IBD stool samples is decreased abundance of Ruminococcaceae (Clostridium cluster IV) and Lachnospiraceae (Clostridium cluster XIVa) [10, 11], which are famous butyrate-producing bacteria [12], compared to stool samples from healthy individuals. Depletion of butyrate in IBD patients might be related to the decreased abundance of butyrate-producing bacteria [13]. Thus, administering either the butyrate-producing bacteria or butyrate itself has been considered as a potential therapeutic agent for the treatment of IBD via its anti-inflammatory effects [14–16].

In spite of the prominent therapeutic potential of butyrate, its actual usage in the clinical setting has been met with scepticism due to the short half-life of butyrate. For instance, when butyrate was intravenously administered to a child with leukaemia (500 mg/kg body weight per day) over a period of several days, limited change in prognosis was made, on the account of the short half-life of butyrate of 6 min, and low peak serum levels of butyrate observed [17]. Similarly, a separate study measured the blood concentrations of butyrate, and showed a rapid elimination following the intravenous injection of arginine butyrate [18]. Moreover, clinically utilizing dietary fibre as major source of butyrate also seems infeasible. The estimated amount of dietary fibre required to observe clinical efficacy for an average individual is 136 g per 1000 kcal, which is approximately 9.7 times more than that of the recommended intake of 14 g per 1000 kcal [19].

In order to overcome this issue of butyrate administration, there has been growing interest in utilizing butyrate derivatives or prodrugs which are more stable and thus more of the administered drug reaches the target site. One of the most prominent prodrugs of butyrate is tributyrin (TB), which is a triglyceride composed of three butyric acid molecules and a glycerol. Butter is one of the richest dietary sources of TB, containing up to 3–4% of its weight [20]. When TB is ingested, for example through consumption of butter, it gets degraded by hepatic lipases releasing 3 butyrate molecules. Thus, dietary intake of TB is expected to yield similar beneficial results as direct consumption of butyrate. A pharmacokinetic study of TB shows that the plasma half-life of butyrate after TB administration is 40 min, which is longer than that of direct butyrate administration [20]. Furthermore, many studies have shown that TB reduces the level of pro-inflammatory cytokines, induces apoptosis of colonic cancer cells similarly to butyrate [21]. In vivo experiment using a rat model of colon carcinogenesis also showed that TB exerts anti-cancer effects [22].

We hypothesized that gut commensal microbes may play a role in TB metabolism and that the resulting metabolites, especially butyrate, may provide health benefits to host. In this research, we screened a metagenomic library constructed using the mouse gut commensal microbiome and identified two novel esterase genes whose products efficiently breakdown TB into butyrate. Finally, we tested the anti-inflammatory effects of the combination of TB and commensal *Escherichia coli* clones that express the esterase genes in dextran sulphate sodium (DSS)–induced colitis mouse model.

## Results

### Tributyrin (TB) degrading clones exhibited butyrate producing capability

**Fig. 1:**
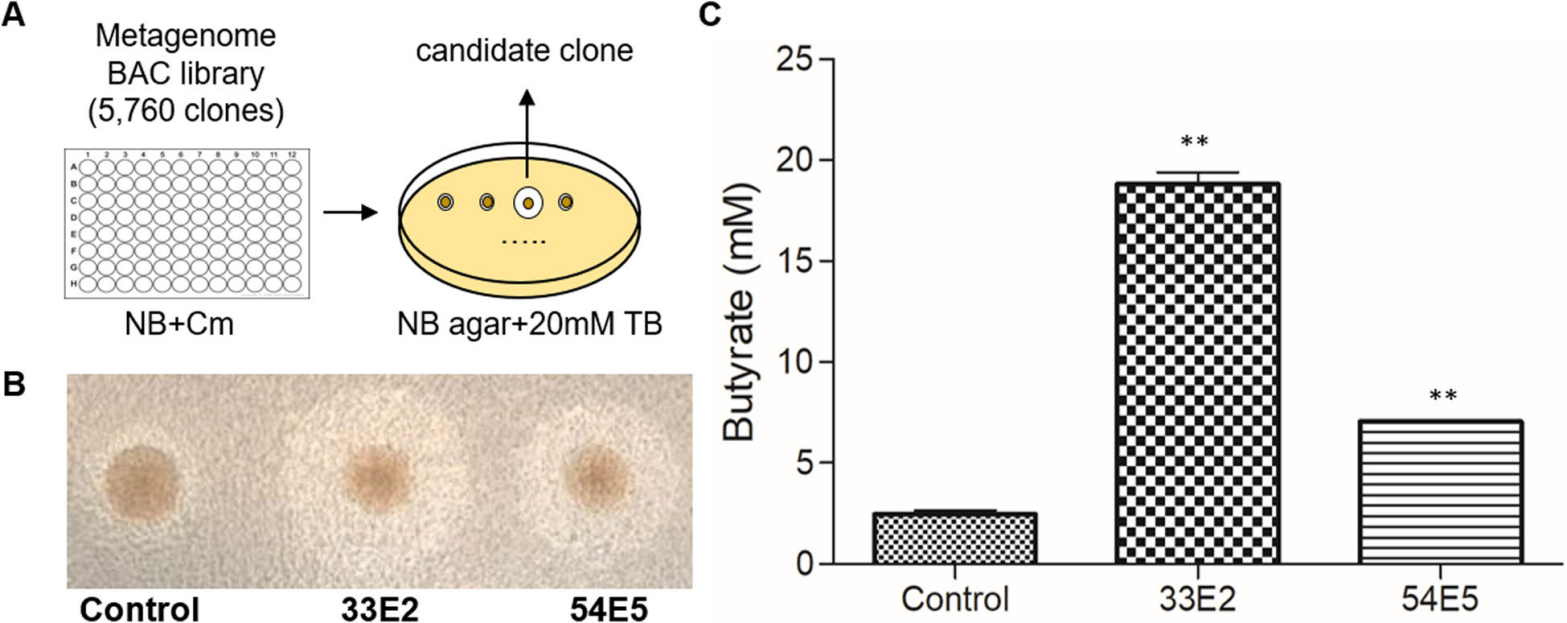
TB degradation of 33E2 and 54E5 clones. **a** Schematic diagram of TB esterase screening of BAC clones. **b** Representative images of a clear zone formation around TB degrading positive clones, 33E2 and 54E5. *E. coli* DH10B containing pIndigoBAC-5 (vector-only control) is also shown as a control. **c** Measurement of butyrate production by 33E2 and 54E5 in the presence of 20 mM of TB. Three independent experiments were performed, and values (means ± SEM) are displayed in each bar. **, *P* < 0.05 compared to Control

Since TB is an ester derived from a glycerol and 3 molecules of butyrate, we postulated that enzymes that can catabolize TB may produce butyrate. Hence, we sought to establish an efficient screening method for high-throughput identification of bacterial clones that express such TB-degrading enzymes. We took advantage of the fact that agar plates containing dissolved TB (Tributyrin Agar, TBA) appear turbid, and that upon TB degradation, the agar turns transparent. Indeed, we observed that bacterial clones producing TB-degrading enzymes, such as PAO1 strain of *Pseudomonas aeruginosa*, form colonies with halos of a cleared zone on TBA (data not shown). Using this simple yet effective screening scheme (Fig. 1a), we screened a metagenomic library consisting of 5760 bacterial artificial chromosome (BAC) clones, where each DNA insert was purified from the gut microbiome of BALB/c mice [23]. An *E. coli* DH10B that contains the pIndigoBAC-5 vector with no insert was used as negative control. Two clones (33E2 and 54E5) exhibited prominent halos around the colony on TBA following 48 h growth (Fig. 1b). These two TB-degrading clones were subsequently isolated and evaluated for butyrate production. Quantitative measurement of butyrate using HPLC implicated that 33E2 and 54E5 produced > 8 times and > 3 times more butyrate, respectively, than the control strain in the presence of 20 mM of the substrate TB (Fig. 1c). This quantitative butyrate measurement is consistent with what was observed on the TB plate (Fig. 1b). In addition, we also confirmed that overnight cultures of 33E2 and 54E5 produced the characteristic smell which signalled butyrate production.

### Sequence analysis of 33E2 and 54E5 reveals the genes responsible for TB degradation

In order to better understand the genes related to TB degradation, the vectors of 33E2 and 54E5 were sequenced and analysed. Consequently, the sequence of 33E2, which contains a 10.4 kb insert, was fully assembled as a complete circular contig. In contrast, the inserted sequence of the 54E5 clone was assembled as three contigs. Based on the lengths of these three contigs, the insert size of the 54E5 clone is estimated to be larger than 64.7 kb. 

When BLASTn searches were performed using the sequences of the 33E2 and 54E5 inserts as query, no significant alignment was retrieved, suggesting that the inserts of these two clones are derived from microbes with unknown genome sequences (Tables 1 and 2). However, the 33E2 insert is most similar to a region of *Blautia producta* genome with 74.3% identity (Table 1). Third, fourth and fifth highest-ranking hits of the BLASTn search were identified as genomic sequences from *Clostridium lentocellum*, *Herbinix* sp. and *Cellulosilyticum* sp. bacteria, respectively, all of which belong to the Lachnospiraceae family [24, 25]. Contig1 and contig3 of the 54E5 insert share regions of high homology with genomic regions of *Duncaniella* sp. (Table 2). No sequence with significant homology to contig2 was detected by BLASTn. Disregarding contig2, most of the hits in Table 2 are associated with members of the Muribaculaceae family [26]. These results suggest that the DNA inserts of 33E2 and 54E5 have originated from bacteria belonging to Lachnospiraceae and Muribaculaceae, respectively. Of note, the predicted origin information of the 33E2 and 54E5 DNA fragments are consistent with the information derived from the BLASTp search of amino acid sequences as query.

**Table 1:** List of BLASTn search results of inserted DNA sequence of 33E2

Contig	Rank^*^	Description	Query Cover	E value	Identity (%)	Accession
33E2	1	*Blautia producta* strain PMF1 chromosome, complete genome	56%	0	74.3	CP035945.1
	2	*Paenibacillus riograndensis* SBR5 genome assembly SBR5(T), chromosome: I	33%	1.00E-80	63.2	LN831776.1
	3	*Clostridium lentocellum* DSM 5427, complete genome	5%	2.00E-34	67.41	CP002582.1
	4	*Herbinix* sp. SD1D genome assembly SD1D, chromosome: I	4%	2.00E-28	67.9	LN879430.1
	5	*Cellulosilyticum* sp. WCF-2 chromosome, complete genome	5%	6.00E-28	66.91	CP034675.1

^*^ Rank of the result is arranged in order of *E*-value

**Table 2:** List of BLASTn search results of inserted DNA sequence of 54E5

Contig	Rank^*^	Description	Query Cover	E value	Identity (%)	Accession
54E5 Contig1	1	*Duncaniella* sp. B8 chromosome, complete genome	9%	0	95.53	CP040121.1
	2	*Duncaniella* sp. C9 chromosome	9%	0	95.53	CP039547.1
	3	Muribaculaceae bacterium DSM 108610 strain Oil-RF-744-WCA-WT-10 chromosome, complete genome	9%	5.00E-133	70.62	CP045696.1
	4	*Muribaculum intestinale* strain YL27 chromosome, complete genome	9%	9.00E-143	87.47	CP015402.2
	5	*Muribaculum intestinale* strain YL27 genome	9%	9.00E-143	87.47	CP021421.1
54E5 Contig2	1	Uncultured bacterium clone NOD_dss_A3_G08 16S ribosomal RNA gene, partial sequence	5%	0	99.93	JQ083840.1
	2	Uncultured bacterium clone WT_ctrl_D1_G10 16S ribosomal RNA gene, partial sequence	5%	0	99.87	JQ084982.1
	3	Uncultured bacterium clone NOD_ctrl_C1iii_C05 16S ribosomal RNA gene, partial sequence	5%	0	99.87	JQ084661.1
	4	Uncultured bacterium clone WT_dss_B5_H07 16S ribosomal RNA gene, partial sequence	5%	0	99.87	JQ084456.1
	5	Uncultured bacterium clone WT_dss_B1_C01 16S ribosomal RNA gene, partial sequence	5%	0	99.87	JQ084160.1
54E5 Contig3	1	*Duncaniella* sp. B8 chromosome, complete genome	34%	0	94.98	CP040121.1
	2	*Duncaniella* sp. C9 chromosome	34%	0	94.98	CP039547.1
	3	*Duncaniella dubosii* strain H5 chromosome	33%	0	89.59	CP039396.1
	4	Uncultured bacterium BAC25G1 genomic sequence	26%	0	95.45	KC595277.1
	5	*Muribaculum intestinale* strain YL27 chromosome, complete genome	34%	0	84.81	CP015402.2

^*^ Rank of the result is arranged in order of *E*-value

**Fig. 2:**
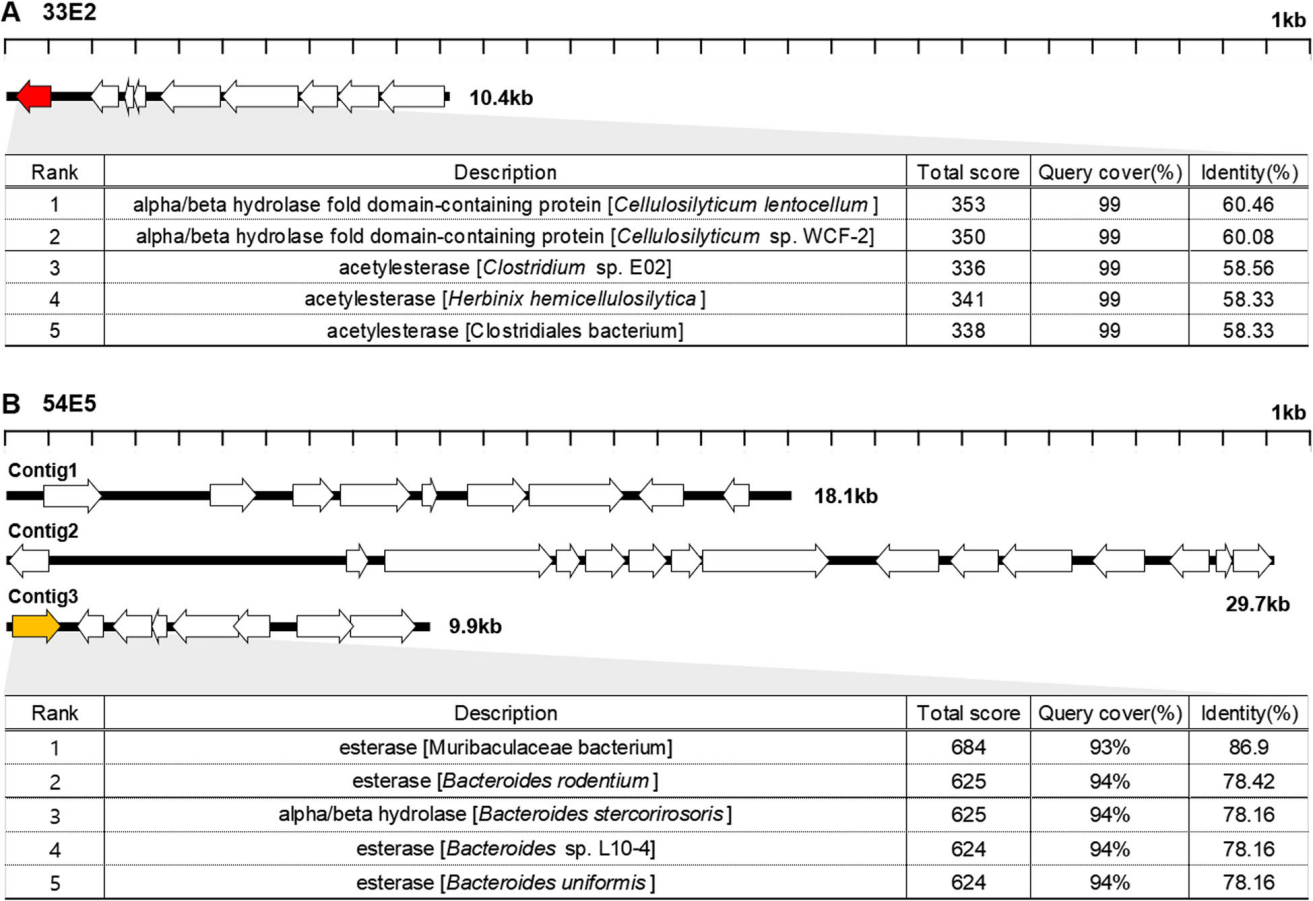
Open reading frame (ORF) maps and BLASTp results of 33E2 and 54E5 clones. ORF maps of the (**a**) 33E2 and (**b**) 54E5 clones**.** The length and direction of the arrows indicate the relative size and direction of each ORF. Detailed information of the ORFs is described in supplementary information. ORFs encoding putative esterases, Tbe1 and Tbe2, are coloured red and orange, respectively, and the top 5 BLASTp hits of the ORFs are listed in the table arranged by increasing *e*-value

The sequence of the 33E2 insert contains 9 ORFs including the first ORF that encodes a putative esterase (Fig. 2a). Although the species origin of the DNA fragment cannot be ascertained, all of the ORFs within the 33E2 insert encode proteins highly homologous to those of Lachnospiraceae family, whose members are well-known producers of short-chain fatty acids [10]. The list of proteins encoded by 33E2 insert genes is provided in Supplementary Information (Table S1). The protein encoded by ORF1 (795 bp, 240 aa) is most homologous to the alpha/beta hydrolase fold domain-containing protein of *Cellulosilyticum lentocellum* with 60.46% amino acid sequence identity. Second-ranked is a protein with the same description and produced by another species of the same genus, *Cellulosilyticum* (Fig. 2a). Other proteins in the list are acetylesterases, either from the genus of *Clostridium* or *Herbinix* (Fig. 2a). These results strongly suggest that TB-degrading capability of the 33E2 clone can be attributed to the enzymatic activity of this gene product.

The sequence of the 54E5 insert contains 32 ORFs and 90.6% (29 ORFs) of the encoded proteins are highly homologous to proteins from the Bacteroidales order (Fig. 2b). The very first gene in contig3 (1194 bp, 397aa) was determined to encode a protein, whose amino acid sequence is highly homologous to a Muribaculaceae bacterium esterase with 86.9% identity. Proteins retrieved from the BLASTp search using this protein as query similarly include esterases or alpha/beta hydrolases from species of the *Bacteroides* genus (Fig. 2b). Hereafter, we named ORF1 of 33E2 and ORF1 of the 54E5 contig1 as *tbe1* and *tbe2*, respectively, with tbe standing for “**t**ri**b**utyrin **e**sterase”.

### Characterization of Tbe1 and Tbe2

**Fig. 3:**
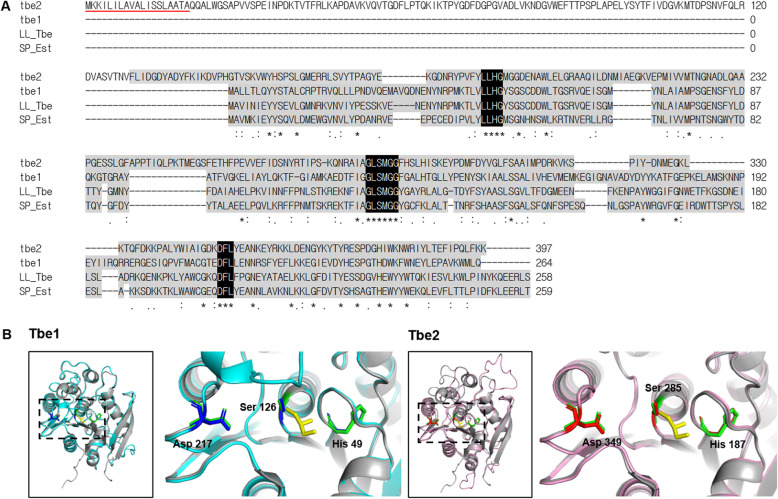
Amino acid sequences and 3-dimensional structures of Tbe1 and Tbe2. **a** Amino acid sequences and multiple amino acid sequence alignment of Tbe1, Tbe2, TB esterase of *L. lactis* (LL_Tbe; UniProtKB Q9L9X0), and esterase of *Streptococcus pneumoniae* (SP_Est; UniProtKB A0A0H2UNZ8). Alpha/beta hydrolase conserved domains and significant consensus sequences are highlighted in grey and black, respectively. Signal peptide is underlined in red. **b** Partial structure of Tbe1 and Tbe2 modelled by SWISS-MODEL. The models are based on the structure of the TB esterase of *Streptococcus pneumoniae* from the RSC Protein Data Bank (2UZ0) as the template. Tbe1, Tbe2, and *S. pneumoniae* TB esterase are shown in cyan, pink, and grey, respectively, with the glycerol residue of TB in yellow. Inside the dotted box is the structure containing the serine-histidine-aspartate (SHD) catalytic triad, as magnified on the right. Each SHD triad of Tbe1, Tbe2 and *S. pneumoniae* tributyrin esterase are shown in blue, red, and green, respectively

In an effort to shed light on the molecular nature of Tbe1 and Tbe2, the respective amino acid sequences were examined using InterPro and SignalP5.0. Analysis of Tbe1 revealed the lack of a signal peptide in the amino acid sequence implying that Tbe1 is probably an intracellular protein. In contrast, Tbe2 amino acid sequence includes a signal peptide sequence, from residue 1 to 19 (Fig. 3a, red underlined), which suggests that Tbe2 may be an extracellular protein. Furthermore, amino acid sequences of Tbe1 and Tbe2 were compared with those of the known TB esterases of *Lactobacillus lactis* (LL_Tbe) and *Streptococcus pneumoniae* (SP_Est) [27, 28]. All of the proteins contained an alpha-beta hydrolase conserved domain (InterPro entry: IPR029058), which is common to hydrolytic enzymes (Fig. 3a). In addition, we also detected the presence of a serine-histidine-aspartate (SHD) catalytic motif, included within the active sites of most alpha-beta hydrolase fold super family proteins [29] (Fig. 3a). Furthermore, all SHD motifs found in our analysis contained 3 consensus sequences (LLHG, GLSMGG, and DFL) (Fig. 3a, highlighted in black).

We then asked whether Tbe1 and Tbe2 proteins are structurally similar to a known esterase. Structures of Tbe1 and Tbe2 were simulated based on the 3D structure of *S. pneumoniae* EstA [28] from the RSCB Protein Data Bank (PDB entry: 2UZ0) as template. Tbe1 shares the overall structural similarity with the template to a greater extent than Tbe2 does (Fig. 3b). However, the SHD triad, found in both Tbe1 and Tbe2 conserved within the catalytic core region, is nicely superimposed with that of the *S. pneumoniae* esterase. These results strongly suggest that Tbe1 and Tbe2 are responsible for TB degradation and butyrate biosynthesis in the 33E2 and 54E5 clones.

### Relative activity of Tbe1 or Tbe2 with the known TB esterases

**Fig. 4:**
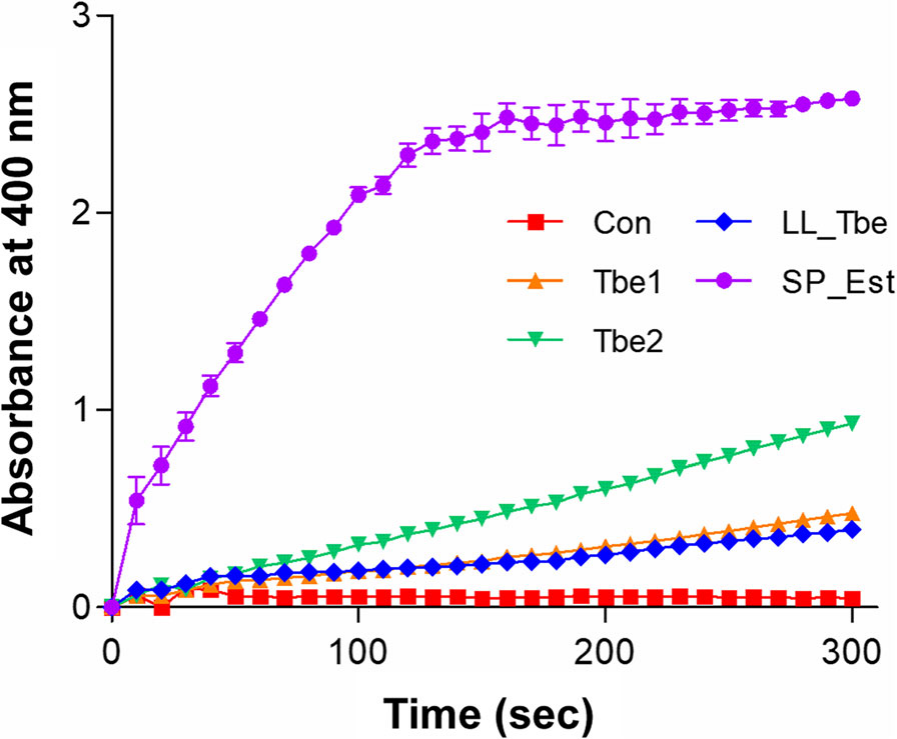
Comparison of enzyme activities of Tbe1 and Tbe2 with LL_Tbe and SP_Est. The t*Ec* cells producing Tbe1 (orange), Tbe2 (green), LL_Tbe (blue) or SP_Est (purple) were examined for the production of *p*-nitrophenol, a breakdown product of *p*-nitrophenol butyrate (*p*-NPB). Bacterial cells were incubated with the enzyme reaction mixture for 5 min and the absorbance at 400 nm was measured every 10 s. The t*Ec* strain harboring the empty pBAD24 plasmid was used as a negative control (red). Three independent experiments were conducted, and values of means ± SEM are displayed in each data point

Next, we compared the enzyme activity of Tbe1 and Tbe2 with previously reported tributyrin esterases [27, 30]. To this end, we further cloned genes encoding the esterase of *Lactococcus lactis* (LL_Tbe) and *Streptococcus pneumoniae* (SP_Est). In order to evaluate the enzyme activities under the same condition, all four genes were cloned into the same location in the pBAD24 plasmid, which is downstream of the promoter sequence of *tbe1*. The bacterial strains were then incubated with 4-nitrophenyl butyrate (*p*-NPB), a substrate of the enzyme. Hydrolysis of *p*-NPB by the esterase enzyme releases 4-nitrophenolate, which can be detected by absorbance at 400 nm. Based on Fig. 4, the esterase of *S. pneumoniae* was the most potent among the four tested enzymes. Of note, Tbe2 is more active than Tbe1, and Tbe1 demonstrated almost identical activity to the *L. lactis* enzyme. While using purified enzymes is more preferable for a precise quantification of the enzyme’s kinetic parameters, these results clearly suggest that Tbe1 and Tbe2, which we identified in the current study, are either as equally as or more active than the esterase of *L. lactis*, a probiotic strain.

### Tbe1 and Tbe2 degrade TB and produce butyrate

**Fig. 5:**
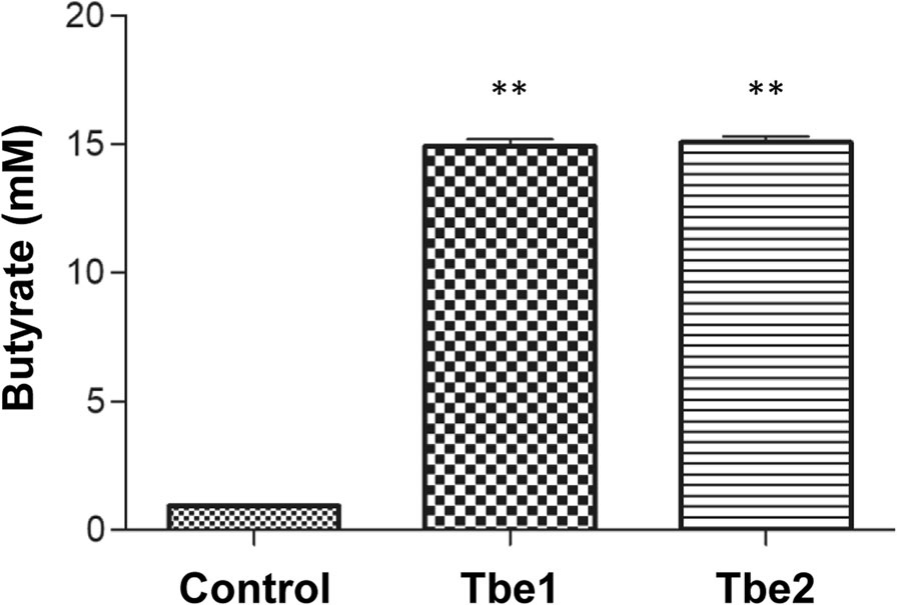
Tbe1 and Tbe2 effectively degrade TB and release butyrate. Measurement of butyrate production by t*Ec* strains harbouring pBAD24::*tbe1* (Tbe1) and pBAD24::*tbe2* (Tbe2) are compared to that of t*Ec* with empty pBAD24 (Control). Bacterial culture was supplemented with 5 mM TB and incubated for 24 h. Three independent experiments were performed, and values (means ± SEM) are displayed in each bar. **, *P* < 0.05 versus Control

Our bioinformatic analyses clearly suggest that Tbe1 and Tbe2 are the most probable proteins contributing to conversion of TB into butyrate. In order to conclusively determine the roles of Tbe1 and Tbe2, we cloned *tbe1* and *tbe2* genes into another *E. coli* host and monitored TB-induced butyrate production. The host strain we used for Tbe expression is the t*Ec* strain that we isolated from the mouse intestines [31]. *tbe1* gene was amplified to include ~ 100 bp sequence upstream of the *tbe1* ORF and cloned into pBAD24 plasmid (pBAD24::*tbe1*). Inclusion of the upstream sequence was intended to enable transcription of *tbe1* using its endogenous promoter. *tbe2* gene was inserted right at the junction of the 54E5 plasmid (Fig. 2b), so we were unable to clone *tbe2* with its endogenous promoter. Therefore, *tbe2* gene was cloned in place of the *tbe1* ORF in pBAD24::*tbe1*, and the resultant plasmid was named pBAD24::*tbe2*. When t*Ec* strains harbouring either plasmids were grown in the presence of 5 mM TB, prominent increases in butyrate production level were observed (Fig. 5). Butyrate production levels in these two cultures were > 16 times higher than the control culture. The characteristic scent of butyrate was again noticed in these two cultures.

Of interest, the level of butyrate produced by *tbe2* gene was almost identical to that by *tbe1* gene. In Fig. 1c, butyrate production by 54E5 was substantially less than that by 33E2. We speculate that this discrepancy was caused by the absence of *tbe2* gene’s own endogenous promoter in the original 54E5 clone. In 54E5, *tbe2* gene expression was probably induced by the upstream sequence part of the pIndigoBAC-5 plasmid. When *tbe2* was transcribed in the presence of the *tbe1* promoter in pBAD24::*tbe2*, greater butyrate production was achieved.

### In vivo on-site butyrate production protects mice against DSS-induced colitis

**Fig. 6:**
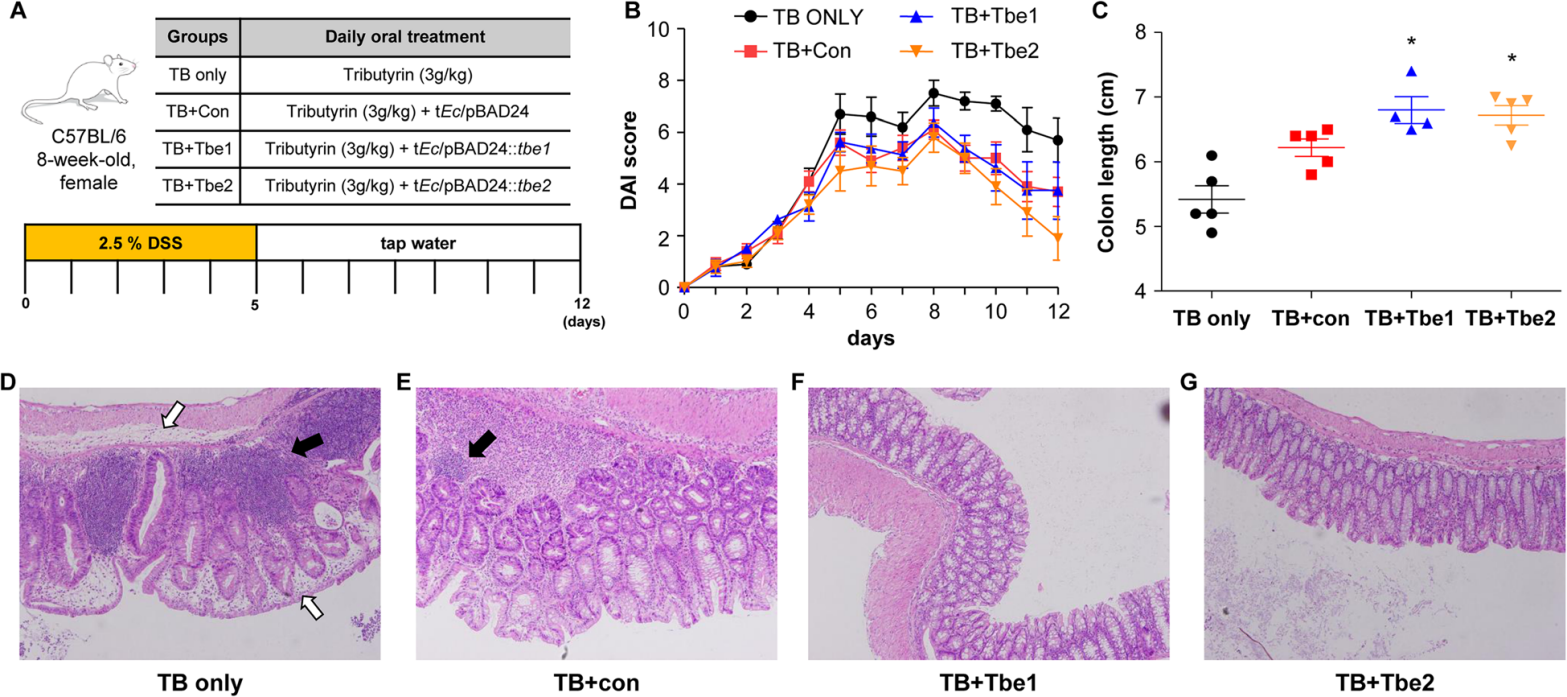
Effects of TB and Tbe on dextran sulphate sodium (DSS)-induced colitis. **a** Schematic diagram of DSS-induced colitis model, **b** disease activity index (DAI) score, and **c** colon length of colitis induced mice treated with only TB (TB ONLY), TB with t*Ec* with empty pBAD24 (TB + Con), t*Ec* with pBAD24::*tbe1* (TB + Tbe1) and t*Ec* with pBAD24::*tbe2* (TB + Tbe2). (D-G) Representative images of H&E stained sections of colons from indicated groups. Arrows denote neutrophil infiltration (black) and edema (white). *, *P* < 0.05 versus TB + Con

Our results so far demonstrate that TB together with Tbe-expressing *E. coli* cells can efficiently produce butyrate, a beneficial microbiome-derived metabolite. To examine whether or not butyrate produced by this system can alleviate intestinal inflammation, we used DSS-induced mouse model of acute colitis. Mice were divided into 4 groups that received different treatments, as illustrated in Fig. 6a. Mice administered 2.5% DSS developed acute colitis characterized by weight loss, bloody diarrhoea, and watery stool, and these outcomes were collectively reflected in the increase of the DAI (Disease Activity Index) score [32] (Fig. 6b). In TB-only group, the DAI scores remained persistently high even after DSS administration was discontinued, suggesting that TB itself did not exert any positive effects toward restoration of a healthy gut condition in mice. It was of particular interest that *E. coli* cells expressing *tbe2* provided significant beneficial effects in mice suffering from severe colitis (Fig. 6b). *E. coli* cells expressing *tbe1* did not seem to confer any protective effects in comparison to the empty vector control group (Fig. 6b). It is not clear why only *tbe2*-expressing *E. coli* cells led to the amelioration of DAI scores.

Another phenotype of the DSS-induced acute colitis is the shortened intestinal length as gut inflammation manifests [33]. On day 12, the last day of the experiment, mice from each group were sacrificed and the lengths of their colons were measured. The colon lengths of mice that received Tbe1- or Tbe2-expressing *E. coli* cells were significantly longer than those of control mice (Fig. 6c), indicating the positive effects of Tbe against DSS-induced colitis. Moreover, the intestinal tissues looked significantly improved by Tbe1 or Tbe2. Hyper-inflammatory responses evidenced by neutrophil infiltration (black arrow) and fluid accumulation (white arrows) were observed in DSS-pretreated mice that received only TB (Fig. 6d). Still higher degrees of neutrophil staining were observed in mice that received control *E. coli* cells (Fig. 6e, black arrow). In contrast, intestinal tissues looked considerably improved when DSS-pretreated mice were treated with TB and *E. coli* cells that express Tbe1 (Fig. 6f) or Tbe2 (Fig. 6g). These results clearly suggest that on-site butyrate production by coupling TB and microbiome-derived novel esterases can exert beneficial effects in the inflamed mouse intestines.

## Discussion

Though computational analysis has enabled significant progress to be made in recent years, functional metagenomic studies using conventional screening methods still remain irreplaceable and invaluable in the identification of novel genes and characterization of their functions. Metagenomic libraries make it feasible to elucidate the functions of genes from unculturable microbes, which has remained a major challenge in gut microbiome studies. For instance, several niche adaptive features of the gut microbiome such as carbohydrate utilization, colonization factors, bile acid resistance and antibiotic resistance have been revealed through the use of metagenomic libraries constructed from the gut commensal microbiomes of either human or other mammals [34–37].

Since dietary fibres are a major source for SCFAs in our gut, many gut metagenome studies have focused on identifying the genes responsible for the metabolism of dietary fibres [38]. Due to the differences in microbiome compositions among individuals, the level of SCFAs produced from dietary fibres varies significantly [39]. Therefore, it would be of crucial benefit to strategize an efficient system of delivering SCFAs in a controlled manner. Here, we established an on-site butyrate production system (OBPS) using a butyrate precursor and microbiome-derived esterases. We chose TB as a prodrug of butyrate and established a functional metagenome screening method that enabled the identification of genes encoding enzymes that specifically catalyse the conversion of TB to butyrate.

The success of our screening method is attributed to the following elements. Firstly, using Triton X-100, we were able to homogenously solidify agar plates that contain highly insoluble TB. TB agar plates acted as an incredibly useful platform, via which high-throughput screening of a large genomic library was performed with minimal plate-to-plate variations. Secondly, the characteristic scent of butyrate aided the differentiation of positive clones from negative ones, especially during the verification stage. Thirdly, TB is a commonly used substrate in esterase assays, yet previous studies have mostly focused on the enzymes rather than the products of the enzymatic reactions. We focused on the fact that TB is an ester of 3 molecules of butyrate condensed with glycerol, and that positive clones would likely generate butyrate as the major product of TB degradation. Fourthly, TB, as a food-grade lipid, can safely be fed to live animals as a prodrug of butyrate, with no expected side-effects. Thus, TB could be safely administered to experimental animals and enabled in vivo confirmation of beneficial effects of TB combined with esterase containing clones.

Until now, TB esterase has gained attention in the context of fermented food and agricultural industries [27, 40, 41]. Here, we report the discovery of novel esterases in the gut, Tbe1 and Tbe2. These esterases were predicted to have originated from two separate phyla; Firmicutes and Bacteroidetes*,* two predominant phyla of the human intestine [42]. Given that these phyla are highly abundant and commonly found across individuals irrespective of geography, race, gender and other characteristics, we speculate that this esterase function serves a physiologically significant role and is inherent across a broad spectrum of microbial species in the intestine.

Interestingly, Tbe1 and Tbe2 share a common catalytic feature. Alpha/beta hydrolase domain is highly conserved among various hydrolytic enzymes [43]. Serine residue of the Ser-His-Asp (SHD) catalytic triad, a typical catalytic motif found in the alpha/beta hydrolase domain, is an essential residue for the enzymatic activity of esterases [30] and often included in the consensus sequence (Gly-x-Ser-x-Gly; x refers to any amino acid) [29]. The amino acid sequences of Tbe1 and Tbe2 both contain the sequence GLSMGG which is found in other known TB esterases. The other two residues, histidine and aspartate, are included in the other two consensus sequences, LLHG and DFL, respectively.

The DNA fragment insert of 33E2 is highly homologous to the genome of Lachnospiraceae. Lachnospiraceae is a gram-positive family that belongs to the Firmicutes phylum. Genera such as *Blautia*, *Coprococcus*, *Dorea*, *Lachnospira*, *Oribacterium*, and *Roseburia* are included in this family. Members of Lachnospiraceae are specialised in utilising plant derived-polysaccharides such as starch, inulin, and arabinoxylan [44]. Similar to *Bacteroides* spp. which have well-established carbohydrate utilization systems encoded within polysaccharide utilization loci (PUL), several members of Lachnospiraceae have been reported to possess similar genomic features termed gram-positive PUL (gpPUL). gpPUL is defined as a collection of genes encoding at least one of 3 components: polysaccharide-degrading enzyme, a carbohydrate transport system and a transcriptional regulator [44]. Moreover, esterases are frequently present within a gpPUL. Some ORFs of 33E2 correspond with the 3 components of gpPUL; ORF7 and ORF8 are predicted as ABC transporter permeases, and ORF9 is a response regulator. Hence, it seems highly possible that Tbe1 of 33E2 is involved in carbohydrate degradation of Lachnospiraceae. The carbohydrate degrading ability of Tbe1 may not be limited to TB, but also be extended to include other various polysaccharides, particularly those that are indigestible by host.

In addition, we examined the effects of TB esterase and TB oral supplementation on the gut barrier function in DSS-induced colitis mouse model. TB delivered via the oral route can be hydrolysed by pancreatic and gastric lipases. Hence, many studies have demonstrated that solely administered TB mediates an anti-inflammatory effect. Mounting evidence suggest that supplementation of TB could induce desirable phenotypes in diet-induced obesity model [45], in piglets [46, 47], and in a murine model of *Clostridium difficile* infection [48]. In this study, we identified microbiome-derived genes encoding active TB esterases and sub-cloned them into an *E. coli* strain with strong intestinal colonization capability [31]. When those strains were delivered together with TB into the mouse intestines, on-site production of butyrate was achieved. More importantly, thus produced butyrate was sufficient for alleviating the inflammatory symptoms in the mouse model of acute colitis.

In the present study, we report the finding of two novel TB esterases, yet our experiments have the following limitations: (i) the limited amount of information regarding TB esterases present in the gut meant that there was no suitable esterase to compare TB degrading activities of the enzymes to; (ii) TB was the only substrate tested in this study and the substrate specificity of Tbe1 and Tbe2 has not yet been fully explored; (iii) given that Tbe1 likely originated from gram-positive Lachnospiraceae, *E. coli* might not be an appropriate surrogate host for Tbe1 expression; (iv) despite strenuous efforts, we were unable to detect consistently elevated levels of butyrate in mouse faecal matters discharged from groups of TB + Tbe1 or TB + Tbe2. We expect that this can be attributed to the rapid metabolism of butyrate by host colonocytes. Further investigations of real-time butyrate production in mouse intestines are required to better rationalize the therapeutic use of OBPS.

Commercially available probiotics are often limited in that the delivered bacterial strains have difficulty stably colonizing the recipient gut and that sufficient amounts of dietary fibre must be available for bacterial cells to exert beneficial effects. However, the OBPS is advantageous in that it is simple, yet the level of butyrate produced can be controlled by the administrator. Therefore, given the abundant evidence for the significance of butyrate in the maintenance of a healthy gut [5–7], the OBPS may prove to be an invaluable treatment option for various diseases.

## Materials and methods

### Bacterial strains and culture conditions

The procedure for BAC (Bacterial Artificial Chromosome) library construction was described in detail elsewhere [23]. Briefly, bacterial DNA was extracted from the combined caecal and colon contents of seven BALB/c mice and digested with HindIII restriction enzyme. The size-selected DNA was cloned into pIndigoBAC-5 (HindIII cloning-ready; Epicentre), and transformed into *E. coli* DH10B. Successfully transformed *E. coli* DH10B was grown in Luria Broth (LB, 10 g tryptone, 5 g NaCl, 5 g yeast extract per litre) supplemented with 25 μg/ml of chloramphenicol (Duchefa Biochemie, Haarlem, Netherlands) at 37 °C. The indigenous non-pathogenic *E. coli* strains t*Ec* (typical *E. coli*) was isolated from CD-1 mouse intestines in a previous study [31]. *Lactococcus lactis* (KCTC 3619) was cultured anaerobically in Brain Heart Infusion (BHI) broth at 37 °C. *Streptococcus pneumoniae* (ATCC 49619) was cultured aerobically in BHI broth at 37 °C. This information is summarised in Table 3.

**Table 3:** Bacterial strains and plasmids used in this study

Strains and plasmids	Relevant characteristic	Source
***E. coli*** **strains**		
DH10B	pIndigoBAC-5 without inserted gene	Laboratory collection
BAC library	DH10B, pIndigoBAC-5 harboring BALB/c mouse gut microbiome DNA	[23]
t*Ec*	wild type strain isolated from CD-1 mouse intestines	[31]
tbe1	t*Ec*, pBAD24::*tbe1*	This study
tbe2	t*Ec*, pBAD24::*tbe2*	This study
***L. lactis***	Type strain KCTC 3619	Laboratory collection
***S. pneumoniae***	Type strain ATCC 49619	Laboratory collection
**Plasmids**		
pIndigoBAC-5	Cp^r^, library backbone vector	Laboratory collection
pBAD24	Amp^r^, cloning vector	Laboratory collection

### Screening of BAC library clones for TB degrading ability

For TB degradation assay, bacteria were grown on TB agar (TBA; 4 g peptone, 3 g NaCl, 3 g yeast extract, 15 g agar, 20 mM TB per litre and 0.02% of triton X-100 as a surfactant) at 37 °C for 48 h. As TB in the media gets degraded by the hydrolytic activity of bacteria, distinct clear zones form around the bacterial colonies.

### Measurement of butyrate

Bacteria were inoculated into 3 ml of TB broth (TBA without agar) within 50 ml conical tubes and incubated at 37 °C for 24 h. TB concentrations used for the initial screening of the metagenomic library and for confirmation of successfully transformed t*Ec* clones were 20 mM and 5 mM, respectively. Bacterial culture supernatants were filtered using 0.2 μm Minisart® Syringe Filters (Sartorius, Germany). Butyrate concentration was measured by high-performance liquid chromatography (HPLC) using Ultimate3000 (Thermo Dionex, USA) with a UV detector (210 nm) and refractive index detector (RefractoMAX520, Japan). For analysis, the Aminex 87H column (300 × 10 mm, Bio-Rad, CA, USA) was used and 0.01 N H_2_SO_4_ (Fluka, USA) were used as an eluent.

### Sequence analysis of the inserted fragments of 33E2 and 54E5

Whole genome sequencing and de novo assembly of BAC plasmids were performed by Macrogen, Inc. (Seoul, Korea). ORFs were detected using the ORFfinder provided by the National Center of Biotechnology Information (NCBI). Sequence homology searches were performed against the NCBI database using BLASTn and BLASTp algorithms. ORFs were confirmed with Prokka [49], and any candidates without significant hits with BLASTp were excluded from analysis. Conserved domain search was done by using InterPro database and signal peptide sequence of protein was predicted based on SignalP 5.0 [50].

### Construction of *E. coli* heterologously expressing *tbe1* and *tbe2*

pBAD24 with an ampicillin resistance marker was used to construct the protein expression vectors (Table 3). The DNA fragment of *tbe1* including the endogenous promoter region (~ 899 bp) was PCR amplified from the 33E2 clone. The DNA fragment of *tbe2* was PCR amplified from 54E5. Since the endogenous promoter region of *tbe2* was not included in the vector of 54E5, the promoter region of *tbe1* was amplified and combined with the amplified fragment of *tbe2* by overlapping PCR (~ 1298 bp). PCR products were cloned into the multi-cloning site of the pBAD24, and the cloned vectors, named pBAD24::*tbe1* and pBAD24::*tbe2*, were transformed into *E. coli* DH10B for amplification. Again, the vectors were extracted from *E. coli* DH10B using AccuPrep Plasmid Mini Extraction kit (Bioneer, Korea) and transformed into t*Ec*. PCR primers used for cloning are listed in Table 4.

**Table 4:** Primers used in this study

Gene name	Direction	Primer sequence (5′ -3′)^a^
*tbe1*_promoter_XmaI	Forward	ACTGCCCGGGAGGTCTCACGGATTCAGGAA
*tbe1*_XbaI	Reverse	ACTCTCTAGATCATTGCAGCATCCATTTAA
*tbe1*_promoter_XbaI	Forward	ACTCTCTAGATTGTAAATAACTCAGATTTGGT
*tbe1*_protmoter_overlapped	Reverse	AATTTTTTTCATATTTTCCTCCTGGTATTGCG
*tbe2*_overlapped	Forward	CAGGAGGAAAATATGAAAAAAATTCTAATCCT
*tbe2*_PstI	Reverse	ACTCCTGCAGTTATTTCTTGAAAAGTTGAG

^a^ Restriction enzyme recognition sequences and overlapped sequences are underlined

### Esterase enzyme activity assay

Tributyrin esterase activity was assessed following procedures described elsewhere [51], using a chromogenic substrate, *p*-nitrophenyl butyrate (*p*-NPB, Sigma Aldrich, USA). Production of *p*-nitrophenol was monitored spectrophotometrically by measuring absorbance at 400 nm. For this assay, t*Ec* transformed with the esterase gene was cultivated for 6 h in LB broth, and then 20 μl aliquot was added to the enzyme reaction mixture of final volume 200 μl.

### Dextran sodium sulphate (DSS) mouse model of acute colitis

Acute colitis was induced in 8-week-old female C57BL/6 mice administered 2.5% dextran sodium sulphate (DSS; molecular weight, 36,000–50,000 Da; MP Biomedicals, OH, USA) in their drinking water for 5 days, which was replaced with normal drinking water for the following 7 days. The mice were monitored daily for body weight, stool consistency and stool bleeding. Disease activity index (DAI) scores were taken in accordance with a previous study [32]. Over the 7-day period following the DSS administration, mice were orally dosed with TB (3 g/kg) combined with the respective treatments. The treatments delivered were PBS, t*Ec*-pBAD24 (empty vector), t*Ec*-pBAD24::*tbe1*, and t*Ec*-pBAD24::*tbe2*, respectively. The dosage of each bacterial clones provided in combination with TB were 1 × 10^8^ CFU. The mice were euthanized by CO_2_ on the 12th day of the experiment and colon lengths were measured.

### Histological analysis

Mouse colons of 3 mm were harvested, opened longitudinall and fixed in 3.7% formaldehyde. Tissues were paraffin-embedded and 5 μm sections were stained with haematoxylin-eosin (H&E). Slides were analysed using an Olympus Microscope (Model. U-LH100HG).

## Supplementary Information

**Additional file 1: Table S1.** Predicted genes in BAC clones.

## Data Availability

The majority of data generated or analyzed during this study are included in this published article or in the supplementary information. The data not shown in the manuscript are available from the corresponding author on reasonable request.

## Abbreviations

UC: Ulcerative colitis
CD: Crohn’s disease
GPR: G-protein-coupled receptor
IBD: Inflammatory bowel disease
SCFA: Short-chain fatty acid
TB: Tributyrin
TBA: Tributyrin agar
BAC: Bacterial artificial chromosome
CFU: Colony forming unit
DSS: Dextran sulfate sodium
Cp: Chloramphenicol
Amp: Ampicillin
Tbe: Tributyrin esterase
SHD triad: Serine-histidine-aspartate triad
OBPS: On-site butyrate production system
PUL: Polysaccharide utilization loci
gpPUL: Gram positive polysaccharide utilization loci
DAI: Disease activity index
