# Supplementary material for: An efficient system for intestinal on-site butyrate production using novel microbiome-derived esterases
Source: J Biol Eng. 2021 Mar 6;15:9. doi: 10.1186/s13036-021-00259-4 (PMC7936488; doi:10.1186/s13036-021-00259-4)
Supplement: Supplementary file 1 — Additional file 1: Table S1. Predicted genes in BAC clones. [file 13036_2021_259_MOESM1_ESM.docx]

**Supporting Information**

**An Efficient System for Intestinal On-site Butyrate Production Using Novel Microbiome-Derived Esterases**

**Dah Hyun Jung, Ji Hyun Yong, Wontae Hwang, Mi Young Yoon and Sang Sun Yoon**

**Table S1. Predicted genes in BAC clones**

| Clone and ORF(s) | Nucleotide range (start:stop, orientation) | Size of putative protein (amino acids) | Putative function (BLAST hit) | Most similar  homologue and Genbank  accession no. | Protein sequence identity |
| --- | --- | --- | --- | --- | --- |
| 33E2, ORF1 | 1088:294 (-) | 240 | alpha/beta hydrolase fold domain-containing protein | *Cellulosilyticum lentocellum* WP_013655742.1 | Identities=159/263(60%) Positives=204/263(77%) |
| 33E2, ORF2 | 2704:2045 (-) | 219 | metallophosphoesterase family protein | Lachnospiraceae bacterium WP_178364842.1 | Identities=145/219(66%) Positives=174/219(79%) |
| 33E2, ORF3 | 3021:2803 (-) | 72 | hypothetical protein | Lachnospiraceae bacterium HBA67762.1 | Identities=47/67(70%) Positives=57/67(85%) |
| 33E2, ORF4 | 3292:3011 (-) | 93 | hypothetical protein | Lachnospiraceae bacterium HBA67762.1 | Identities=46/79(58%) Positives=58/79(73%) |
| 33E2, ORF5 | 5083:3665 (-) | 472 | hypothetical protein | Lachnospiraceae bacterium WP_178780199.1 | Identities=377/472(80%) Positives=428/472(90%) |
| 33E2, ORF6 | 6885:5116 (-) | 589 | extracellular solute-binding protein | unclassified Lachnospiraceae WP_178780201.1 | Identities=494/592(83%) Positives=536/592(90%) |
| 33E2, ORF7 | 7842:6946 (-) | 298 | ABC transporter permease subunit | Lachnospiraceae bacterium WP_178780203.1 | Identities=272/298(91%) Positives=288/298(96%) |
| 33E2, ORF8 | 8783:7863 (-) | 306 | ABC transporter permease subunit | Lachnospiraceae bacterium WP_178780205.1 | Identities=275/306(90%) Positives=294/306(96%) |
| 33E2, ORF9 | 10338:8890 (-) | 482 | response regulator | Lachnospiraceae bacterium WP_178780207.1 | Identities=302/479(63%) Positives=387/479(80%) |
| 54E5_contig1, ORF1 | 887:2212 (+) | 441 | anaerobic sulfatase-maturase | uncultured Ruminococcus sp. SCH69572.1 | Identities=114/404(28%) Positives=191/404(47%) |
| 54E5_contig1, ORF2 | 4713:5810 (+) | 365 | IS110 family transposase | Dysgonomonas gadei WP_006801250.1 | Identities=155/363(43%) Positives=224/363(61%) |
| 54E5_contig1, ORF3 | 6662:7618 (+) | 318 | IS30 family transposase | Muribaculaceae bacterium WP_123541742.1 | Identities=318/318(100%)  Positives=318/318(100%) |
| 54E5_contig1, ORF4 | 7732:9411 (+) | 559 | hypothetical protein | Prevotella timonensis WP_103002866.1 | Identities=263/552(48%) Positives=372/552(67%) |
| 54E5_contig1, ORF5 | 9620:9982 (+) | 120 | hypothetical protein | Clostridia bacterium WP_176935933.1 | Identities=50/119(42%)  Positives=73/119(61%) |
| 54E5_contig1, ORF6 | 10716:12053 (+) | 445 | MULTISPECIES: radical SAM protein | Bacteroidales WP_123483718.1 | Identities=223/424(53%) Positives=306/424(72%) |
| 54E5_contig1, ORF7 | 12050:14293 (+) | 747 | hypothetical protein | Porphyromonas endodontalis WP_004332090.1 | Identities=335/727(46%) Positives=463/727(63%) |
| 54E5_contig1, ORF8 | 15593:14499 (-) | 364 | tRNA (N6-isopentenyl adenosine(37)-C2)-methylthiotransferase MiaB | Porphyromonadaceae bacterium HAP29096.1 | Identities=305/358(85%) Positives=331/358(92%) |
| 54E5_contig1, ORF9 | 17106:16567 (-) | 179 | transposase | Bacteroidales bacterium WP_177817021.1 | Identities=154/178(87%)  Positives=169/178(94%) |
| 54E5_contig2, ORF1 | 1005:1 (-) | 335 | hypothetical protein | Muribaculaceae bacterium GFI39866.1 | Identities=200/222(90%) Positives=214/222(96%) |
| 54E5_contig2, ORF2 | 8001:8516 (+) | 171 | outer membrane beta-barrel protein | Rikenellaceae bacterium WP_178214144.1 | Identities=73/154(47%) Positives=101/154(65%) |
| 54E5_contig2, ORF3 | 8882:12865 (+) | 1327 | DUF2723 domain-containing protein | unclassified Muribaculaceae WP_123407644.1 | Identities=876/1326(66%) Positives=1035/1326(78%) |
| 54E5_contig2, ORF4 | 12897:13520 (+) | 207 | polysaccharide deacetylase family protein | Muribaculaceae bacterium WP_123482617.1 | Identities=177/206(86%) Positives=194/206(94%) |
| 54E5_contig2, ORF5 | 13609:14613 (+) | 334 | NAD-dependent epimerase/dehydratase family protein | Bacteroidales bacterium WP_178233412.1 | Identities=238/332(72%) Positives=280/332(84%) |
| 54E5_contig2, ORF6 | 14610:15536 (+) | 308 | DUF4271 domain-containing protein | Bacteroidales WP_123482615.1 | Identities=158/312(51%) Positives=223/312(71%) |
| 54E5_contig2, ORF7 | 15600:16373 (+) | 257 | uroporphyrinogen-III synthase | Bacteroidales WP_123482621.1 | Identities=196/250(78%) Positives=226/250(90%) |
| 54E5_contig2, ORF8 | 16373:19393 (+) | 1006 | phosphoenolpyruvate synthase | Bacteroidales WP_123482614.1 | Identities=826/990(83%) Positives=903/990(91%) |
| 54E5_contig2, ORF9 | 21846:20353 (-) | 497 | succinate CoA transferase | Bacteroidales WP_123482612.1 | Identities=378/493(77%) Positives=432/493(87%) |
| 54E5_contig2, ORF10 | 23236:22091 (-) | 381 | outer membrane beta-barrel protein | unclassified Prevotella WP_172176428.1 | Identities=138/389(35%) Positives=207/389(53%) |
| 54E5_contig2, ORF11 | 24979:23312 (-) | 555 | hypothetical protein | Bacteroidales bacterium WP_178261924.1 | Identities=152/315(48%) Positives=213/315(67%) |
| 54E5_contig2, ORF12 | 26676:25426 (-) | 416 | 4Fe-4S cluster-binding domain-containing protein | Chryseobacterium sp. WP_089028128.1 | Identities=153/404(38%) Positives=235/404(58%) |
| 54E5_contig2, ORF13 | 28199:27222 (-) | 325 | IS110 family transposase | Bacteroidales WP_123481028.1 | Identities=324/325(99%) Positives=324/325(99%) |
| 54E5_contig2, ORF14 | 28383:28754 (+) | 123 | transposase family protein | unclassified Muribaculaceae WP_123612963.1 | Identities=123/123(100%) Positives=123/123(100%) |
| 54E5_contig2, ORF15 | 28768:29694 (+) | 308 | hypothetical protein | Muribaculaceae bacterium GFI39866.1 | Identities=298/308(97%) Positives=303/308(98%) |
| 54E5_contig3, ORF1 | 51:1244 (+) | 397 | esterase | Muribaculaceae bacterium WP_172481901.1 | Identities=341/397(86%) Positives=367/397(92%) |
| 54E5_contig3, ORF2 | 2343:1687 (-) | 218 | transposase | unclassified Muribaculaceae WP_123612964.1 | Identities=217/218(99%) Positives=217/218(99%) |
| 54E5_contig3, ORF3 | 3448:2522 (-) | 308 | hypothetical protein | Muribaculaceae bacterium GFI39866.1 | Identities=299/308(97%) Positives=304/308(98%) |
| 54E5_contig3, ORF4 | 3833:3462 (-) | 123 | transposase family protein | unclassified Muribaculaceae WP_123612963.1 | Identities=122/123(99%) Positives=122/123(99%) |
| 54E5_contig3, ORF5 | 5393:4005 (-) | 462 | NADPH-dependent glutamate synthase | Bacteroidales WP_123481547.1 | Identities=412/462(89%)  Positives=434/462(93%) |
| 54E5_contig3, ORF6 | 6240:5386 (-) | 284 | sulfide/dihydroorotate dehydrogenase-like FAD/NAD-binding protein | Bacteroidales WP_123481546.1 | Identities=242/285(85%) Positives=266/285(93%) |
| 54E5_contig3, ORF7 | 6811:8145 (+) | 444 | Trk system potassium transporter TrkA | Bacteroidales bacterium WP_177925523.1 | Identities=228/446(51%) Positives=304/446(68%) |
| 54E5_contig3, ORF8 | 8142:9605 (+) | 487 | TrkH family potassium uptake protein | Bacteroidales bacterium WP_177910348.1 | Identities=257/496(52%) Positives=355/496(71%) |
